# How to Keep the Baby Well

**Published:** 1890-11

**Authors:** 


### HOW TO KEEP THE BABY WELL.

The infant's stomach does not readily accommodate itself to changes in the diet, and regularity in quantity, quality and temperature is one of the cardinal rules in feeding babies. Not until the child is nearly a year old, should it be allowed to make digestive experiments upon table food, and very little, if any, meat should be given to very young children. At this time it may be weaned, if at the breast, and given some light form of nourishment, such as broth, gruel, egg and Carnrick's food. Not until it has all its teeth, or when it is two years of age, should it be allowed to come to the table or partake of diet prepared for its elders. Prior to four months of age, it has very little power for digesting starchy foods, and consequently, food of this kind is very apt to cause diarrhœa ; so that the rule is, when this occurs to abstain from all kinds of starch until the child is at least four months old. This does not apply to malted milk, which only contains milk-sugar, nor to Carnrick's food, in which the carbohydrates are all changed into dextrin and soluble starch, which does not irritate, and is not readily fermentable.

In addition to feeding, the child should have water to drink between meals. The water should be boiled and kept free from contamination of all kinds. Where there is a tendency to bowel disorder, a little gum arabic, rice, or barley may be boiled with the water, which should afterwards be carefully strained. This is usually well borne, and may be given freely.

A word should be said with regard to keeping the bottles absolutely clean. After each feeding, the remnant of the milk in the bottle should be discarded, and the bottle immediately washed in hot water, with a pinch of bicarbonate of sodium, or with ashes, but not with shot, which has caused lead poisoning in infants. Several bottles should be in use at one time so that one may be always clean. The nipple should be of black or pure rubber, and not of the white or vulcanized rubber. It should fit over the top of the bottle, and all kinds of nursing bottles with tubes should be rejected. A little coarse salt will assist in cleansing the rubber when it becomes coated, and the nipples should be kept in clean water, containing a pinch of soda, in the intervals of using them.—*Baltimore Health Journal.*

